# Supplementary material for: Chromothripsis during telomere crisis is independent of NHEJ, and consistent with a replicative origin
Source: Genome Res. 2019 May;29(5):737–49. doi: 10.1101/gr.240705.118 (PMC6499312; doi:10.1101/gr.240705.118)
Supplement: Supplemental Material [file supp_gr.240705.118_Supplemental_file_1.zip › contigs/annotated_contigs/DB86/contig.2.DB86_length_666_mean_cov_8.52552552553.docx]

**DB86_length_666_mean_cov_8.52552552553**

```
GCCCTCCCCCCGGCCCCCCCCACGAGGCCCGCCGCCCCTCCCCACCC|CCCATGGACCCCACATACTGCCCCCGACTCCCCACAATGCC
                                                >chr9:137655619-137655905 - E=3e-157
CCCAGACCCCACCACGCTGCCCCTGGACCCCCACAACAACCCTTGAGGGCCAGGCTTGCTCCTGTAGGCAGGCTCAGACTTCCTCCTGG

AGTTGCCAGCCAGCCTCGCCTGTGCCTCCTCTCTTTGGCTTCCCTGGGAAATGCCCAGGTCCGTCTGTTCTCAGGTCGAGCTCTCTCTC

TTCTAAGCTCCTGGCTGATCCCAGATCCTGCTCCAGCAGGAGCCTTGGACAAAGCCTTTCTCCACAC|GTTT|CTTTGGGAGGCCAAGG
                                                                        >chr9:140805805
CAGGTGGATCCCCTGAGGTCAGGAGTTCGAGACCAGCCTGACCAACATGGTGAAACCCCGTCTCTACAAAAAAAAATACAAACACTAGC
-140806134 - E=3e-183
TGGGCGCAGTAGCGCATGCCTGTAATTCCAGCTACTCGGGAGGCTGAGGCAGGAGAACTGCTTGAGCCCAGGAGGCAGAGGTTGCAGTG

AGCCGAGATTGCGCCATTGCACTCCAGTCTGGGCTACAGAGCGAGACTCCGACTCGAATAAATAAATAAAGAAAAGATAAATATCTCAA

AATAAAAAAAGAAAAAAAGGACAATAAAAACCCTTCTATTCAAACC
```
